# Comprehensive analysis of mitogen-activated protein kinase cascades in chrysanthemum

**DOI:** 10.7717/peerj.5037

**Published:** 2018-06-19

**Authors:** Aiping Song, Yueheng Hu, Lian Ding, Xue Zhang, Peiling Li, Ye Liu, Fadi Chen

**Affiliations:** 1 College of Horticulture, Nanjing Agricultural University, Key Laboratory of Landscape Agriculture, Ministry of Agriculture, Nanjing, Jiangsu, China; 2 College of Horticulture, Xinyang Agricultural and Forestry University, Xinyang, Henan, China

**Keywords:** *Chrysanthemum morifolium*, Mitogen-activated protein kinase, Phylogenetic analysis, Protein-protein interaction, Stress and development response

## Abstract

**Background:**

Mitogen-activated protein kinase (MAPK) cascades, an important type of pathway in eukaryotic signaling networks, play a key role in plant defense responses, growth and development.

**Methods:**

Phylogenetic analysis and conserved motif analysis of the MKK and MPK families in *Arabidopsis thaliana*, *Helianthus annuus* and *Chrysanthemum morifolium* classified *MKK* genes and *MPK* genes. qRT-PCR was used for the expression patterns of *CmMPK* and *CmMKK* genes, and yeast two-hybrid assay was applied to clear the interaction between CmMPKs and CmMKKs.

**Results:**

We characterized six *MKK* genes and 11 *MPK* genes in chrysanthemum based on transcriptomic sequences and classified these genes into four groups. qRT-PCR analysis demonstrated that *CmMKK*s and *CmMPK*s exhibited various expression patterns in different organs of chrysanthemum and in response to abiotic stresses and phytohormone treatments. Furthermore, a yeast two-hybrid assay was applied to analyze the interaction between CmMKKs and CmMPKs and reveal the MAPK cascades in chrysanthemum.

**Discussion:**

Our data led us to propose that CmMKK4-CmMPK13 and CmMKK2-CmMPK4 may be involved in regulating salt resistance and in the relationship between CmMKK9 and CmMPK6 and temperature stress.

## Introduction

Mitogen-activated protein kinase (MAPK) cascades are a ubiquitous signal transduction pathway that is widely distributed in eukaryotes, transferring and amplifying external signals by the phosphorylation of downstream proteins (Hamel et al., 2006; Kazuya et al., 2002). The typical MAPK cascade pathway consists of MAPKKK-MAPKK-MAPK. MAPKKKs, as key molecules downstream of receptor-like protein kinases (RLKs), can be activated by protein phosphorylation (Wang et al., 2015). The MAPKs ultimately activated transduce extracellular environmental signals to cells by activating downstream response factors such as kinases, enzymes and transcription factors, regulating a series of intracellular reactions such as cell growth, differentiation, apoptosis and stress response (Ren et al., 2008).

MAPKK is a bispecific protein kinase that is activated by phosphorylation of the threonine and tyrosine residues of the TXY motif in the MAPK activation loop. According to the conserved S/T-xxxxx-S/T sequence and the D site, these motifs can be divided into Groups A, B, C and D (Kazuya et al., 2002). The MAPK protein family is a promiscuous family of serine/threonine kinases that phosphorylates a variety of substrates, including transcription factors, protein kinases and cytoskeleton-related proteins (Zhang et al., 2014).

Mitogen-activated protein kinase is reported not only to be widely involved in the transduction of plant stress signals in activating the expression of stress-resistance genes (Kong et al., 2013) but also to participate in signaling associated with plant growth and development (Xu & Zhang, 2015). OsMKK6-OsMPK3 is a signaling pathway that responds to cold stress in rice (Xie, Kato & Imai, 2012). The GhMKK4-GhMPK20-GhWRKY40 cascade in cotton plays important roles in the pathogenesis of *Fusarium oxysporum* (Wang et al., 2018). Plant growth and development require precise coordination among cells, tissues and organs. In eukaryotes, cell–cell and cell-environment communication usually involves cell surface receptors (Xu & Zhang, 2015). The MKK7-MPK6 signaling pathway regulates polar auxin transport to determine shoot branching in *Arabidopsis thaliana* (Jia et al., 2016). YODA and MPK6 were found to participate in embryonic root development in *A. thaliana* via growth regulation and cell division orientation (Smékalová et al., 2014). The YDA-MKK4/MKK5-MPK3/MPK6 cascade regulates local cell proliferation downstream of the endoplasmic reticulum (ER) receptor, which shapes plant organ morphology (Meng et al., 2012). In addition, the ER-YDA pathway regulates an immune surveillance system conferring broad-spectrum disease resistance (Sopeña-Torres et al., 2018).

Chrysanthemum (*Chrysanthemum morifolium*), one of the four most famous cut flowers in the world, is in great annual demand worldwide and is susceptible to various biotic and abiotic stresses (An et al., 2014). The MAPK cascade genes have been investigated in model plants, including Arabidopsis, rice and tomato, but rarely characterized in chrysanthemum. Here, we isolated six *MKK*s and 11 *MPK*s in chrysanthemum based on a set of transcriptomic data. We performed a comparative phylogenetic analysis of MPKs and MKKs in *A. thaliana*, *Helianthus annuus* and *C. morifolium* genes in silico and investigated the transcription pattern of *CmMPK*s and *CmMKK*s in different organs of chrysanthemum, as well as in response to various phytohormones and abiotic stresses, using qRT-PCR. A yeast two-hybrid assay was applied to analyze the interactions between CmMKKs and CmMPKs. We found that CmMKKs and CmMPKs participated widely in plant stress responses and plant growth, laying the foundation for future research on the function of *CmMPKs* and *CmMKKs* in *C. morifolium*.

## Materials and Methods

### Plant materials and growth conditions

Cuttings of the *C. morifolium* cultivar “Jinba” were obtained from the Chrysanthemum Germplasm Resource Preservation Center (Nanjing Agricultural University, Nanjing, China), then rooted in vermiculite without fertilizer in a greenhouse. After 14 days, the cuttings were transplanted to their corresponding growth substrates and then subjected to a range of stress and plant hormone treatments.

### Database searches and sequencing of full-length *CmMPK* and *CmMKK* cDNAs

Arabidopsis MPK and MKK protein sequences were downloaded from The Arabidopsis Information Resource (TAIR) database and used as query sequences to identify *CmMPK* and *CmMKK* genes in chrysanthemum. Multiple alignments among the identified *CmMPK* and *CmMKK* sequences were also performed to avoid repetition. Finally, seventeen pairs of gene-specific primers (Table S1) were designed to amplify the full open reading frame (ORF) sequences. The related amplicons were purified using an AxyPrep DNA Gel Extraction Kit (Axygen, Hangzhou, China) and ligated into pMD19-T (Takara, Tokyo, Japan) for sequencing.

### Phylogenetic tree construction and sequence analysis

A phylogenetic tree was constructed with MEGA version 7.0 using the Maximum Likelihood method (Kumar, Stecher & Tamura, 2016). Multisequence alignments of CmMPK and CmMKK protein sequences were performed among *A. thaliana*, *H. annuus* (Badouin et al., 2017) and *C. morifolium* using MUSCLE (Edgar, 2004). The theoretical isoelectric point (PI) and molecular weight (MW) of CmMPK and CmMKK proteins were calculated using the Compute PI/MW online tool (http://web.expasy.org/compute_pi/), and PSORT online tool was used to predict their subcellular localization. The MEME v4.10.2 program (Bailey et al., 2015) was employed to identify the motifs present in the CmMPK and CmMKK proteins.

### Plant treatments

The transcription profiles of *CmMPK* and *CmMKK* genes in roots, stems, unexpanded leaves, mature leaves and senescent leaves were explored. Various abiotic stresses were imposed, including high salinity (200 mM NaCl) and osmotic stress (20% w/v polyethylene glycol 6000, PEG 6000) (Song et al., 2012). For the NaCl and PEG 6000 assays, young plants were transferred to liquid medium containing the stress agent, and the second true leaves were sampled at various time points (Song et al., 2014a). Other seedlings were subjected to a period at either 4 °C or 40 °C in a chamber with 50 μmol·m^−2^·s^−1^ of light, the second true leaves were sampled after 1 h treatment (Song et al., 2014c). The phytohormone treatments involved spraying the leaves with either 50 μM abscisic acid (ABA), or 5 mM gibberellins (GA) (Song et al., 2014b). The plants were sampled at 0 h and after 1 h treatments. After sampling, all of the collected material was snap frozen immediately in liquid nitrogen and stored at −70 °C before RNA extraction. Three biological replicates per experiment were performed.

### Real-time quantitative PCR

Total RNA was isolated from samples using the Quick RNA isolation Kit (Huayueyang, Beijing, China), with the RNase-free DNase I treatment to remove potential genomic DNA contamination. The first cDNA strand was synthesized from 1 μg of total RNA using PrimeScript^™^ RT reagent Kit with gDNA Eraser (Takara, Tokyo, Japan) according to the manufacturer’s instructions. qPCR was performed using a Mastercycler ep realplex instrument (Eppendorf, Hamburg, Germany). The qPCR reaction cocktail and cycling regime were applied as described by Song et al. (2016). Gene-specific primers (sequences shown in Table S1) were designed using Primer Premier 5, and the *EF1*α gene was employed as a reference. Relative transcript abundances were calculated via the 2^−ΔΔCT^ method (Livak & Schmittgen, 2001). A total of three independent experiments were conducted.

### Data analysis

For expression pattern analysis, the relative transcript expression levels of each *CmMPK* and *CmMKK* were log2 transformed, and the profiles were compared using Cluster v3.0 software (Hoon et al., 2011) and visualized using Treeview (Eisen et al., 1998). A one-way analysis of variance, followed by the use of LSD test (*P* = 0.05), was employed to statistical analysis.

### Yeast two-hybrid assay

For the yeast two-hybrid assay, the *CmMKK* ORF fragments were cloned into the pGADT7 vector in-frame with the GAL4 activation domain (primers given in Table S1). The *CmMPK* ORF cDNA fragments were cloned into the pGBKT7 vector in-frame and proximal to the binding domain (primers refer to Table S1). The pairs of pGADT7-CmMKK and pGBKT7-CmMPK vectors were cotransformed into the Y2H yeast strain using the Matchmaker Gold Yeast Two-Hybrid System (Clontech, Mountain View, CA, USA). Positive clones were plated onto selective SD/-Leu/-Trp medium. A total of 2 days later, monoclones were picked and suspended in 200 μL of water, and 4 μL of the suspension was placed on SD/-Leu/-Trp/-His/-Ade medium and on SD/-Leu/-Trp/-His/-Ade/X-a-gal medium. The results could be observed after 2 days. Three independent experiments were conducted.

## Results

### Phylogenetic relationships among MPK and MKK proteins of *A. thaliana*, *H. annuus* and *C. morifolium*

Details regarding the isolated 11 CmMPK (GenBank: MG334201–MG334212) and six CmMKK (GenBank: MG334196–MG334201) sequences are provided in Table 1. The CmMKK and CmMPK proteins were predicted to have different subcellular localizations, including the chloroplast, cytoskeleton, cytoplasm, mitochondrion, or nucleus, based on PSORT analysis.

**Table 1:** Summary of CmMPK/CmMKK sequences and the identity of likely *A. thaliana* homologs.

Gene	GenBank accession no.	Amino acids length (aa)	AtMKK orthologs	Locus name	PI	MW	Subcellular loclization
*CmMKK2*	MG334196	354	*AT4G29810.1*	MAP kinase kinase 2	5.47	38,986.44	cyto
*CmMKK3*	MG334197	517	*AT5G40440.4*	Mitogen-activated protein kinase kinase 3	4.88	57,585.41	cyto, nucl
*CmMKK4*	MG334198	360	*AT3G21220.2*	MAP kinase kinase 5	9.37	40,537.26	chlo, nucl
*CmMKK5*	MG334199	371	*AT3G21220.2*	MAP kinase kinase 5	9.24	41,049.59	chlo, mito, nucl
*CmMKK6*	MG334200	334	*AT5G56580.1*	MAP kinase kinase 6	6.37	37,432.26	nucl, cyto
*CmMKK9*	MG334201	312	*AT1G73500.1*	MAP kinase kinase 9	8.03	35,245.67	cyto, nucl
*CmMPK1*	MG334202	379	*AT1G10210.3*	Mitogen-activated protein kinase 1	6.64	43,816.73	cyto, cysk
*CmMPK3.1*	MG334203	371	*AT3G45640.1*	Mitogen-activated protein kinase 3	5.39	42,568.67	cysk, cyto, nucl
*CmMPK3.2*	MG334204	371	*AT3G45640.1*	Mitogen-activated protein kinase 3	5.34	42,670.78	cysk, cyto, nucl
*CmMPK4.1*	MG334205	379	*AT4G01370.1*	MAP kinase 4	6.13	43,628.62	cyto, cysk
*CmMPK4.2*	MG334206	387	*AT4G01370.1*	MAP kinase 4	6.28	44,200.39	cysk, chlo, nucl, cyto
*CmMPK6*	MG334207	391	*AT2G43790.1*	MAP kinase 6	5.44	45,151.47	nucl, cyto
*CmMPK9.1*	MG334208	507	*AT3G18040.4*	MAP kinase 9	6.43	57,992.35	cyto, cysk, nucl, chlo
*CmMPK9.2*	MG334209	538	*AT3G18040.3*	MAP kinase 9	6.03	61,502.68	cyto, chlo, nucl
*CmMPK13*	MG334210	381	*AT1G07880.2*	Protein kinase superfamily protein	5.81	43,434.13	chlo, cyto, nucl
*CmMPK16*	MG334211	563	*AT5G19010.1*	mitogen-activated protein kinase 16	9.13	63,910.22	cyto, chlo, nucl
*CmMPK18*	MG334212	599	*AT1G53510.1*	mitogen-activated protein kinase 18	9.41	68,021.07	cyto, nucl

**Note:**

PI, isoelectric point; MW, molecular weight; chlo, chloroplast; cysk, cytoskeleton; cyto, cytoplasm; mito, mitochondrion; nucl, nucleus.

**Figure 1:**
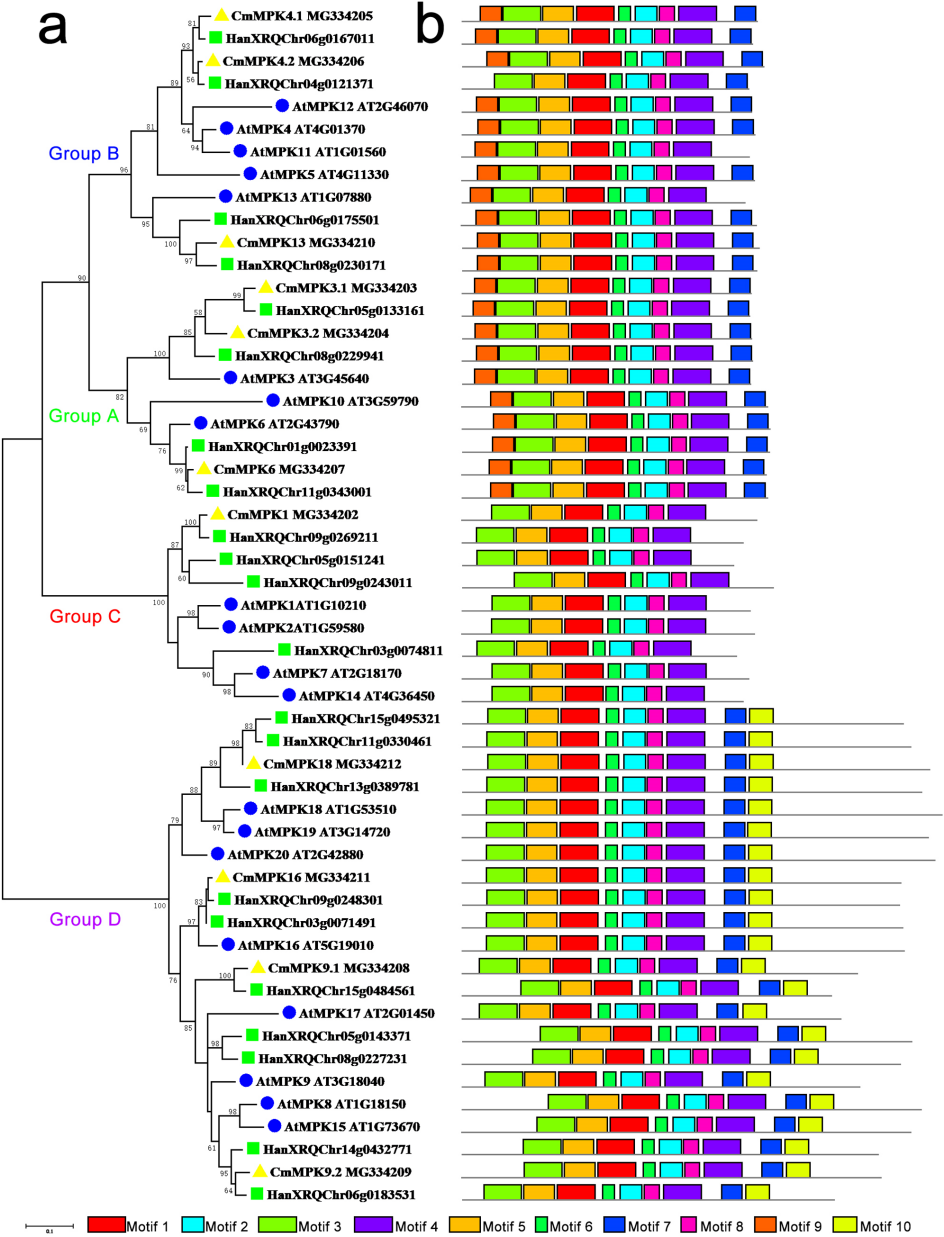
Phylogenetic and domain analyses of MPKs. (A) Phylogenetic relationships of *A. thaliana*, *H. annuus* and *C. morifolium* MAPK. The phylogenetic tree was constructed by MUSCLE using the MEGA7.0 program. (B) Schematic diagram of the amino acid motifs of *A. thaliana*, *H. annuus* and *C. morifolium* MPKs. Motif analysis was performed using MEME 4.0 software as described in the methods. The black solid line represents the corresponding MPK and its length. The different-colored boxes represent different motifs and their positions in each MPK sequence. The blue circle: *A. thaliana*; the green square: *H. annuus*; the yellow triangle: *C. morifolium*.

The phylogenetic tree constructed for the MPK protein family in *A. thaliana*, *H. annuus* and *C. morifolium* could be divided into four groups: A, B, C and D (Fig. 1). The motifs of Group A were similar to those of Group B, while motif 10 was unique to Group D. Group C possessed the fewest motifs and lacked motif 7, which was present in the other groups. The major members of Group A were MPK3/6/10. MPK3 had two paralogs in *C. morifolium* and *H. annuus*, whereas only one ortholog was found in *A. thaliana*, which had another member, MPK10, in this group. The major members of Group B were MPK4/5/11/12/13. MPK4 was similar to MPK3, and motif 9 was missing from *H. annuus* HanXRQChr04g0121371 in this group, while MPK5/11/12 was unique to Arabidopsis. The major members of Group C were MPK1/2/7/14 in *A. thaliana*, but *C. morifolium* had only one ortholog of MPK1, while *H. annuus* had three MPK1/2 homologs. In Group D, *C. morifolium* MPK9.1 was separate from MPK9.2, and MPK18/19/20 was found in both *A. thaliana* and *H. annuus*, while only MPK18 was found in *C. morifolium*. Only one copy of MPK16 was present in *A. thaliana* and *C. morifolium*, while two members could be found in *H. annuus*.

**Figure 2:**
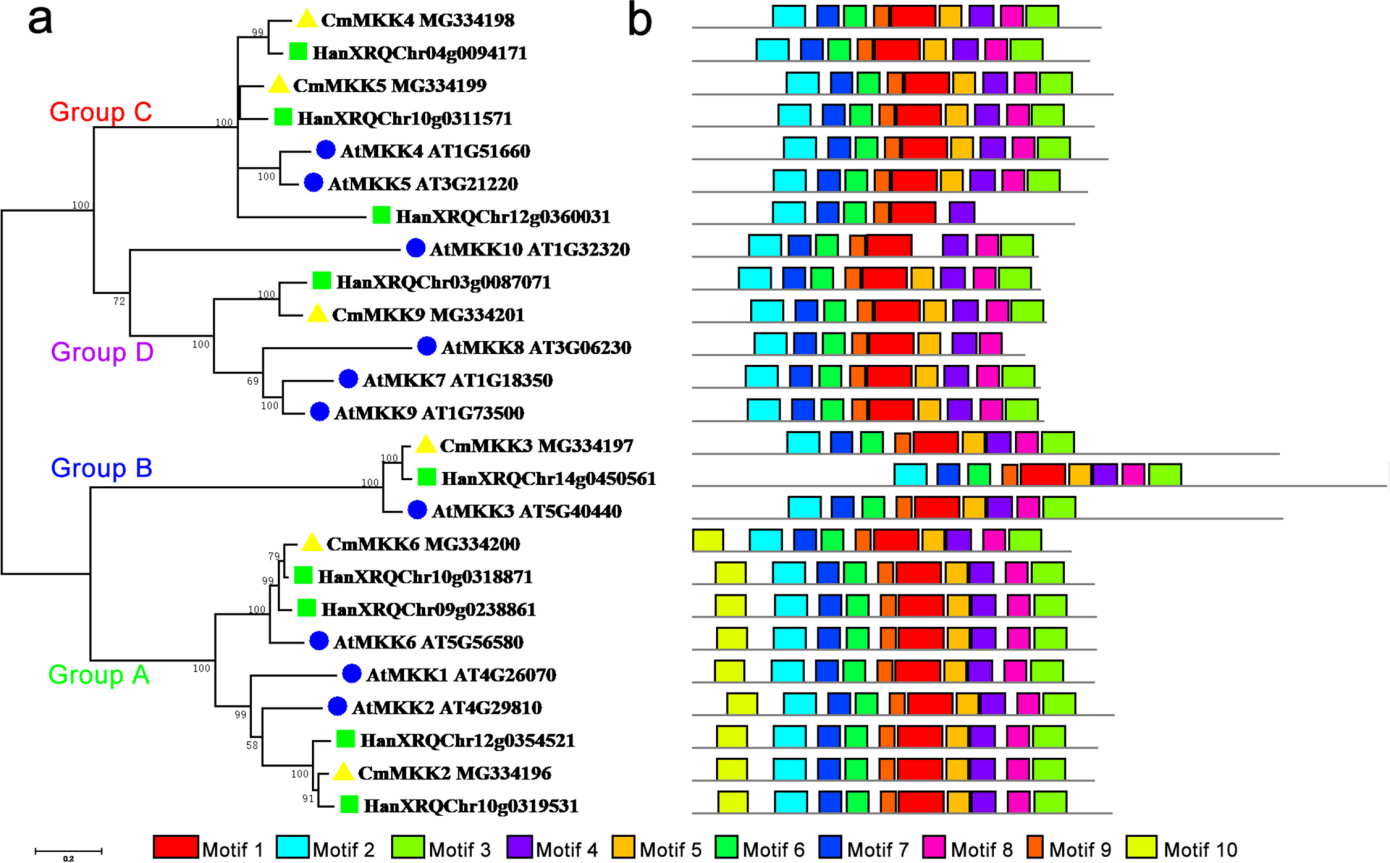
Phylogenetic and domain analyses of MKKs. (A) Phylogenetic relationships of *A. thaliana*, *H. annuus* and *C. morifolium* MKKs. The phylogenetic tree was constructed by MUSCLE using the MEGA7.0 program. (B) Schematic diagram of amino acid motifs of *A. thaliana*, *H. annuus* and *C. morifolium* MKKs. Motif analysis was performed using MEME 4.0 software as described in the methods. The black solid line represents the corresponding MKK and its length. The different-colored boxes represent different motifs and their positions in each MKK sequence. The blue circle: *A. thaliana*; the green square: *H. annuus*; the yellow triangle: *C. morifolium*.

The phylogenetic tree of the family of MKK proteins in *A. thaliana*, *H. annuus* and *C. morifolium* could also be divided into four groups: Groups A, B, C and D (Fig. 2). Group A contained motif 10, which could not be found in any other three groups, although the other three groups contained similar motifs. The major members of the Group A were MKK6/1/2, while MKK6 was the only ortholog in *A. thaliana* and *C. morifolium*, and *H. annuus* had two paralogs. Group B contained only MKK3, and there was only one ortholog in the species. The main members of Group C were MKK4 and MKK5, but one more member, HanXRQChr12g0360031, was present in *H. annuus*, which instead was missing motif 9 and motif 3 from this group. The major members of Group D were MKK7/8/9/10. Only MKK9 was found in *H. annuus* and *C. morifolium*, while MKK7/8/10 were unique to *A. thaliana,* and MKK10 in *A. thaliana* was lacking motif 5.

### Differential expression patterns of *CmMPK* and *CmMKK* genes in different organs

**Figure 3:**
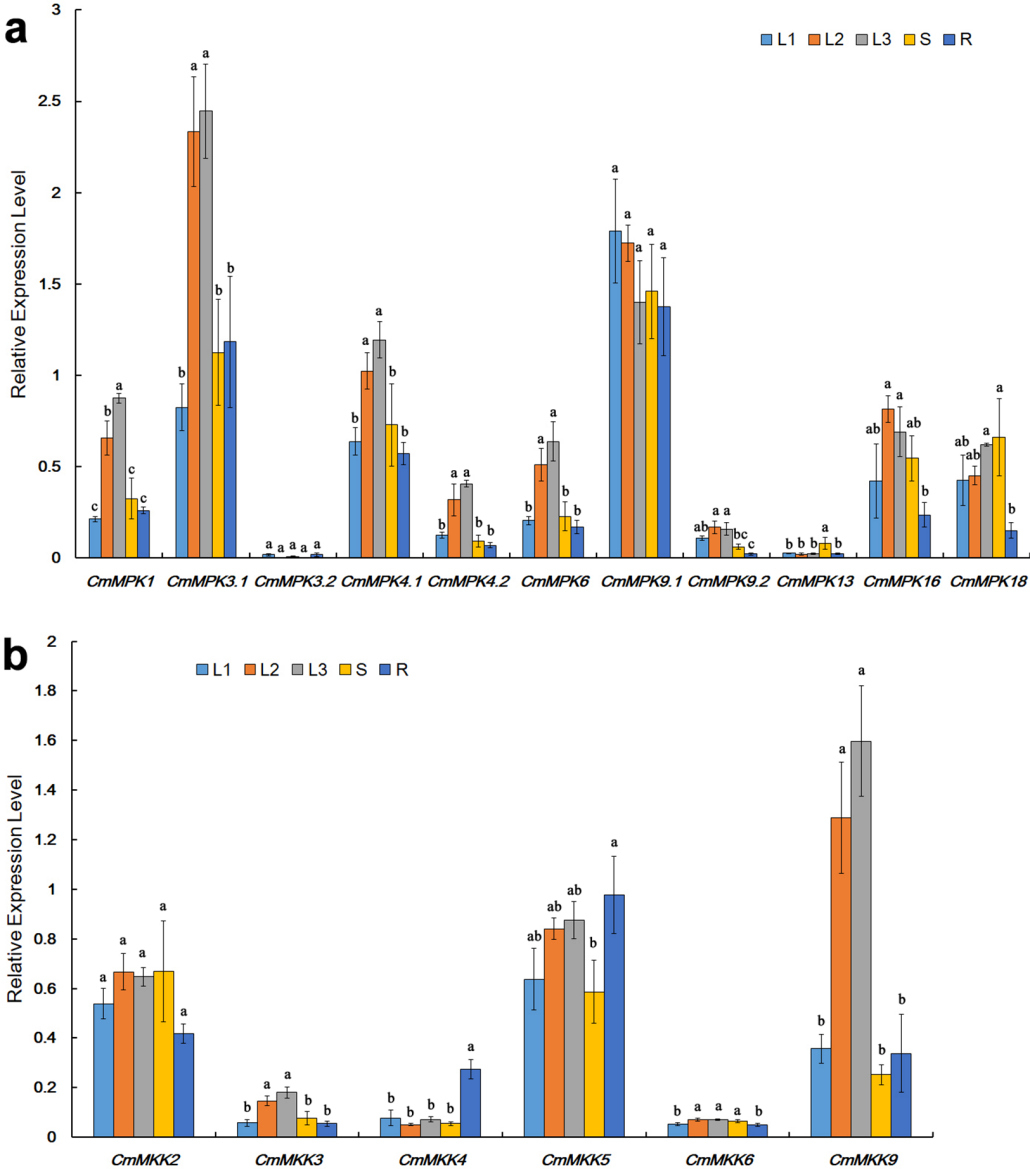
Expression patterns of *CmMPKs* (A) and *CmMKKs* (B) in different organs/tissues obtained by qRT-PCR analysis. L1: unexpanded leaves; L2: mature leaves; L3: senescent leaves; S: stems; R: roots. All samples were run in triplicate, and the data were normalized relative to *EF1a*.

The expression patterns of *CmMPK*s in various organs, including unexpanded leaves, mature leaves, senescent leaves, stems and roots (Fig. 3A), were quantitatively analyzed. The results showed that compared to those in unexpanded leaves, the expression levels of *CmMPK1*, *CmMPK3.1*, *CmMPK4.1*, *CmMPK4.2*, *CmMPK6* and *CmMPK16* in mature leaves and senescent leaves were significantly increased (Fig. 3A). Some pairs of paralogs, such as *CmMPK3.1* and *CmMPK3.2*, *CmMPK4.1* and *CmMPK4.2*, and *CmMPK9.1* and *CmMPK9.2,* had different expression levels and expression trends in different organs. The expression levels of *CmMPK3.2*, *CmMPK9.2* and *CmMPK13* in the whole plant were lower than those of other *CmMPK*s. Quantitative analysis of *CmMKK* expression levels in different organ showed that the expression of *CmMKK9* was significantly increased in mature and senescence leaves, while *CmMKK4* was highest in the roots (Fig. 3B).

### Differential responses of the *CmMPKs* and *CmMKKs* to abiotic stress

**Figure 4:**
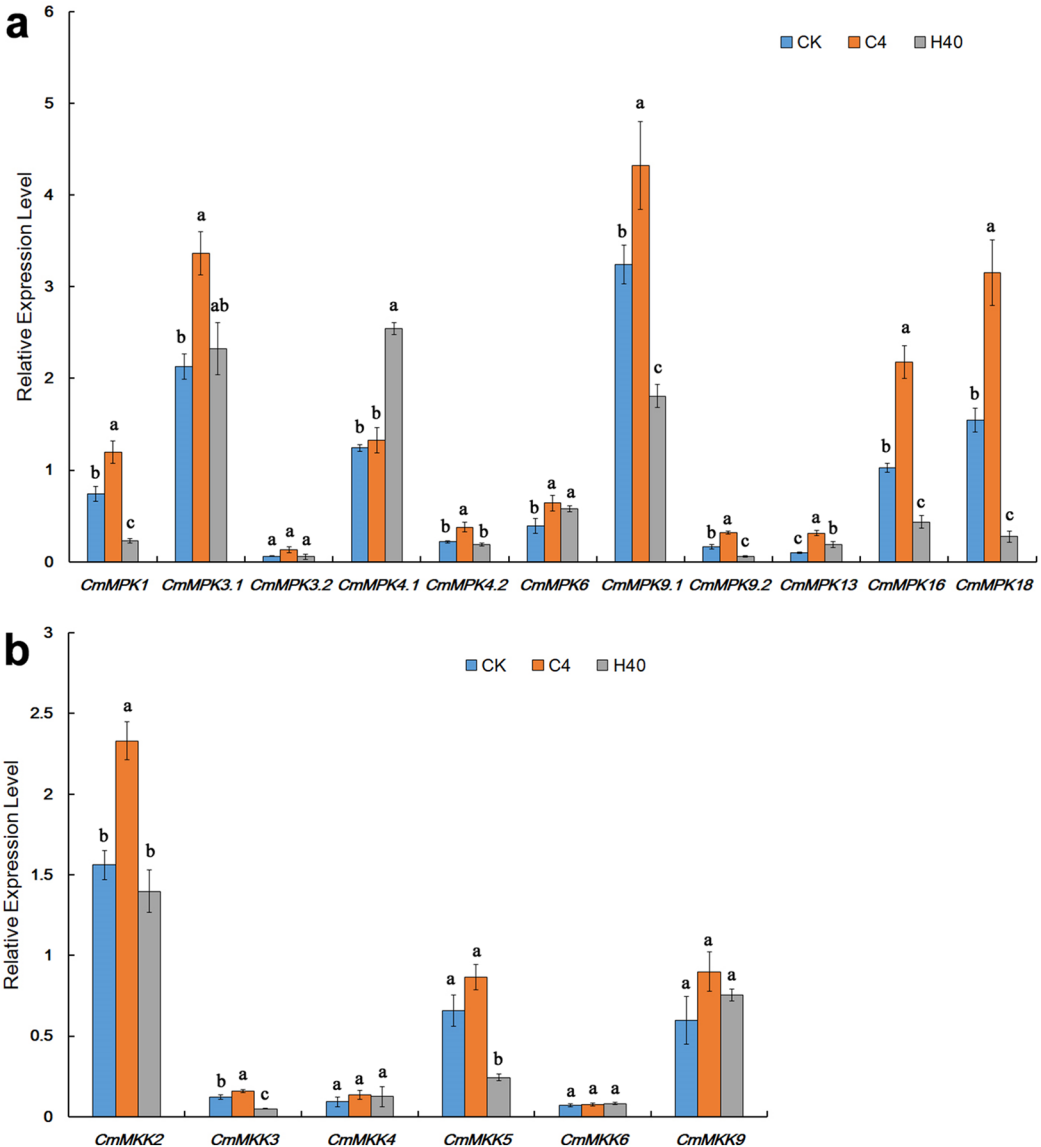
Expression patterns of *CmMPKs* (A) and *CmMKKs* (B) under cold and heat shock treatment in chrysanthemum obtained by qRT-PCR analysis. CK: control plants; C4: the plants treated with 4 °C; H40: the plants treated with 40 °C. Details of the treatments are reported in “Materials and Methods”. All samples were run in triplicate, and the data were normalized relative to *EF1a.*

After heat shock and cold treatment for 1 h, the expression levels of *CmMPKs* were quantitatively analyzed (Fig. 4A). As shown, we found that *CmMPK3.1*, *CmMPK3.2* and *CmMPK4.2* were induced by cold treatment and did not respond to heat shock. *CmMPK1*, *CmMPK9.1*, *CmMPK9.2*, *CmMPK16* and *CmMPK18* were all induced by cold treatment, and their expression levels decreased after heat shock treatment. *CmMPK4.1* was induced after heat shock treatment but did not respond to cold stress. *CmMPK6* and *CmMPK13* responded to cold and heat shock treatment, but the expression levels after heat shock treatment were slightly lower than those after cold treatment.

After heat shock and cold treatment for 1 h, the expression levels of *CmMKKs* were quantitatively analyzed (Fig. 4B). We found that *CmMKK2*, *CmMKK3* and *CmMKK5* all showed increased expression in response to cold, while their expression levels decreased after heat shock treatment. *CmMKK9* responded to both cold and heat shock treatment, but the expression level after heat shock treatment was slightly lower than that after cold treatment. *CmMKK4* and *CmMKK6* did not respond to cold heat shock treatment.

**Figure 5:**
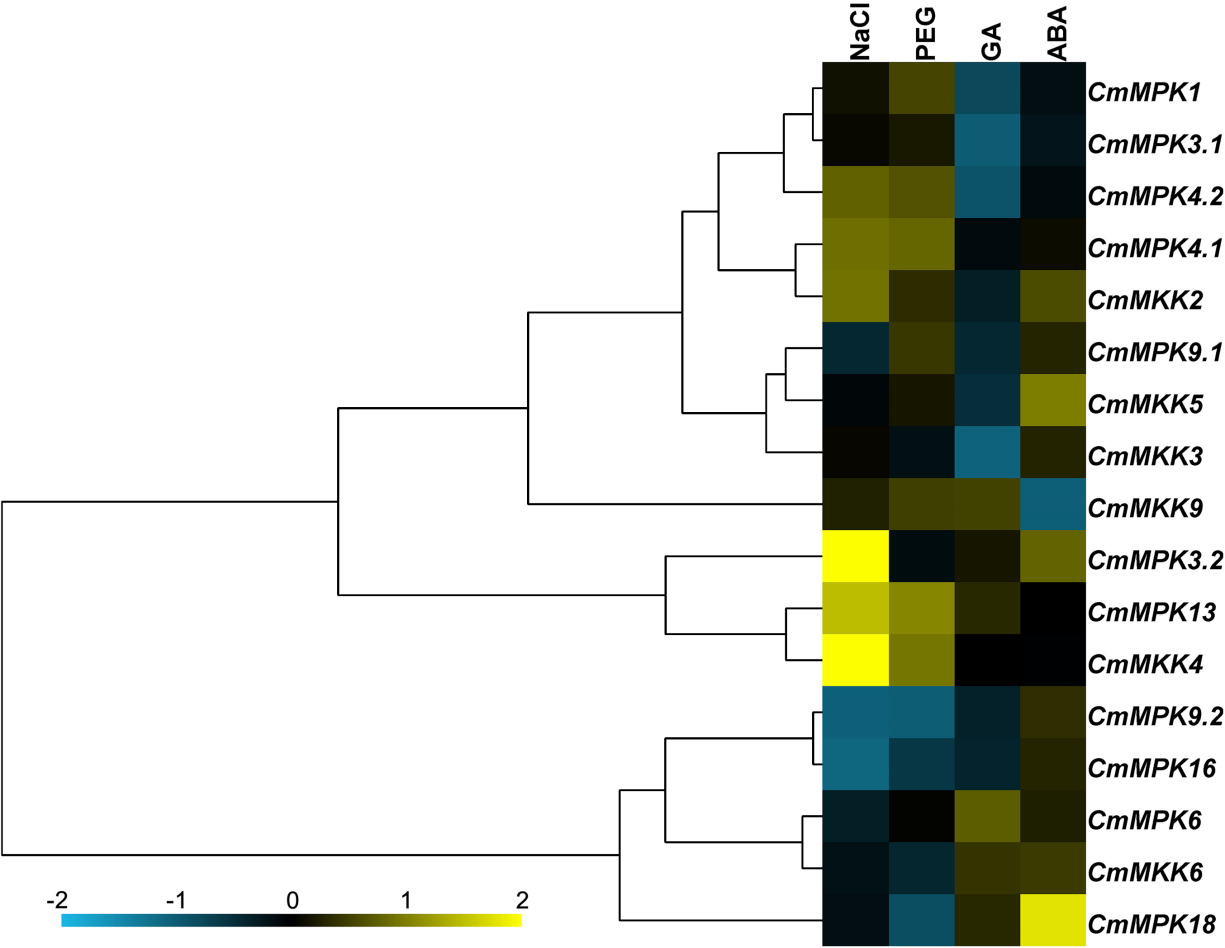
Expression patterns of *CmMPKs* and *CmMKKs* under abiotic stress treatments in chrysanthemum obtained by qRT-PCR analysis, and shown as a heatmap. The details of the treatments are reported in “Materials and Methods”. All samples were run in triplicate, and the data were normalized relative to *EF1a.*

The expression patterns of 11 *CmMPKs* and six *CmMKKs* after two abiotic stresses and two exogenous hormone treatments were analyzed by qRT-PCR (Fig. 5). The results showed that *CmMPK3.2*, *CmMPK13* and *CmMKK4* were induced after NaCl treatment, while the expression levels of *CmMPK9.2* and *CmMPK16* were significantly decreased. The expression of *CmMPK18* increased significantly after ABA treatment, while the expression of *CmMKK6* decreased significantly. *CmMPK4.1* and *CmMPK4.2* had similar expression patterns, but the expression patterns of two other pairs of paralogs, *CmMPK3.1* and *CmMPK3.2* and *CmMPK9.1* and *CmMPK9.2*, showed differences. The expression patterns of *CmMKK2* and *CmMPK4.1* were similar, as were those of *CmMKK5/CmMPK9.1*, *CmMKK4/CmMPK13* and *CmMKK6/CmMPK16*.

### The interaction between CmMPKs and CmMKKs in the yeast two-hybrid assay

**Figure 6:**
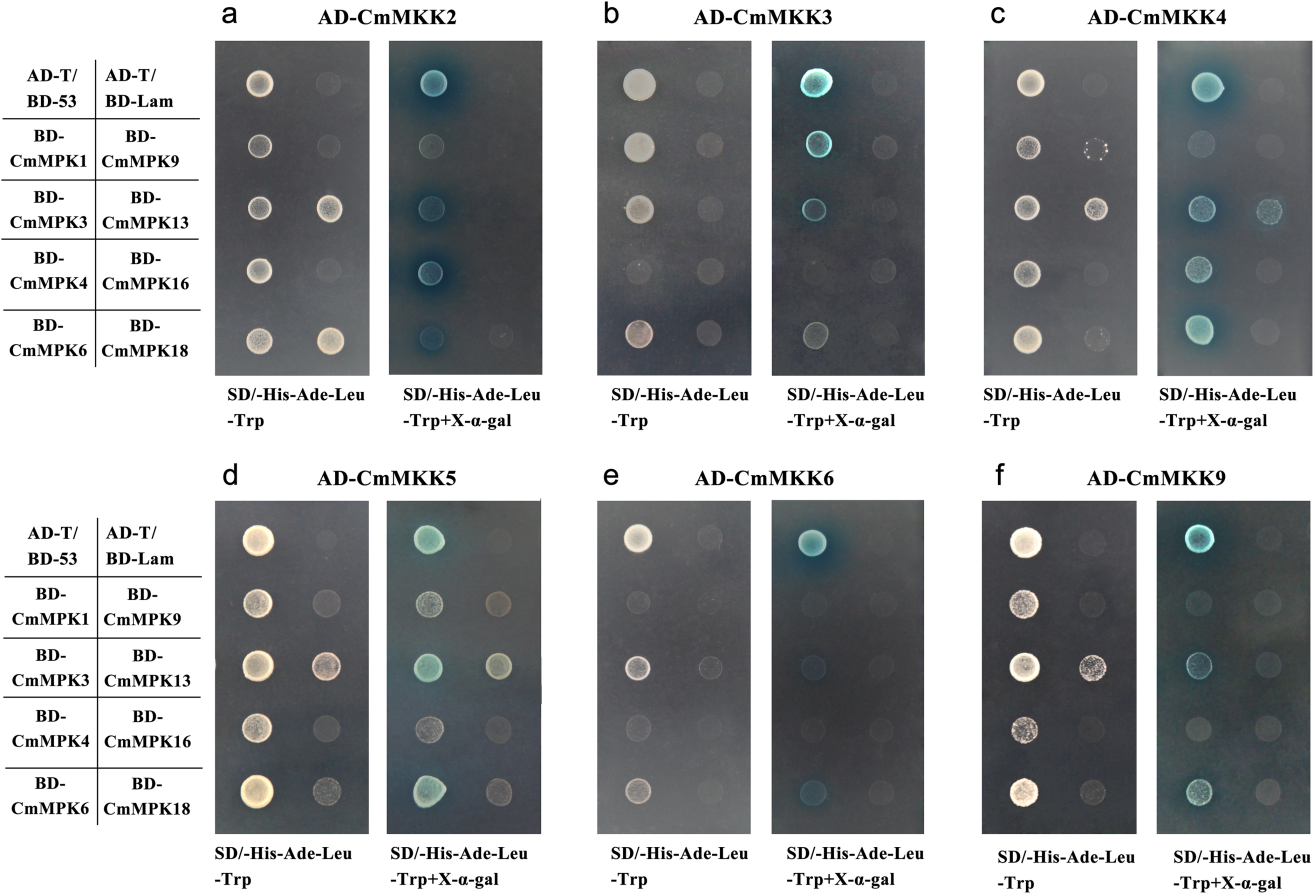
Interactions of CmMKKs with CmMPKs in yeast. The CmMKK ORF fragments were cloned into the pGADT7 (AD) vector in-frame with the GAL4 activation domain, while the CmMPK ORF cDNA fragments were cloned into the pGBKT7 (BD) vector in-frame with the GAL4 binding domain. pGADT7-T-CmMKKx was used as bait, and pGBKT7-CmMPKs were used as prey; (A) pGADT7-CmMKK2; (B) pGADT7-CmMKK3; (C) pGADT7-CmMKK4; (D) pGADT7-CmMKK5; (E) pGADT7-CmMKK6; (F) pGADT7-CmMKK9. In addition, pGADT7-T/pGBKT7-53 was used as a positive control, while pGADT7-T/pGBKT7-Lam was used as a negative control.

The yeast two-hybrid assay was applied to discover the interactions between CmMPKs and CmMKKs. As shown in Table S2 and Fig. 6, all CmMKKs could interact with CmMPK1/3/6. CmMPK4 interacted strongly with CmMKK2 and weakly with CmMKK4/5. CmMPK9/16 did not interact with any CmMKKs, CmMPK13 interacted weakly with CmMKK2/4/5/6, and CmMPK18 interacted only with CmMKK2.

## Discussion

### Comparative analysis of the *A. thaliana*, *H. annuus* and *C. morifolium* MPK and MKK gene families

In this study, 11 *CmMPK* and six *CmMKK* genes were isolated in chrysanthemum based on transcriptome data and classified according to their sequence similarity. To elucidate the phylogenetic relationships among the *CmMPK* genes and to infer the evolutionary history of the gene family, we constructed a phylogenetic tree with high support rates using MPK members from *A. thaliana*, *H. annuus* and *C. morifolium.* The MPKs were well divided into four groups, and we found that most of the motifs of MPKs in the same group were similar to each other. MPK3 has been reported to play roles in abiotic resistance (Beckers et al., 2009) (Li et al., 2017), ovule development (Wang et al., 2008) and anther development (Hord et al., 2008). Only one MPK3 was found in *A. thaliana*, but two paralogs were found in both *H. annuus* and *C. morifolium* (Fig. 1). Additionally, the expression patterns of *CmMPK3.1* and *CmMPK3.2* were significantly different in different plant organs (Fig. 3A) and in response to temperature stresses (Fig. 4A) and other abiotic stresses (Fig. 5). This difference suggested that *MPK3* was duplicated and that functional differentiation occurred after the speciation event that divided *A. thaliana* and the other two species. The same situation was also found in *MPK4* (Group B) and *MPK9* (Group D). MPK4 was reported to be required for light-induced anthocyanin accumulation (Li et al., 2016) and to be associated with cold resistance (Du et al., 2017). Notably compared to the other members of MPK4, one of the MPK4 proteins in *H. annuus* missed motif 9, and the functions of the two MPK4 paralogs in *H. annuus* were suspected to be differentiated. MPK9 was reported to be involved in stomatal closure in *A. thaliana* (Khokon et al., 2017). There were significant differences in the expression patterns between *CmMPK9.1* and *CmMPK9.2* in various organs of *C. morifolium* (Fig. 3A) and in response to temperature stress (Fig. 4A) and other abiotic stress treatments (Fig. 5). This evidence suggested that the functions of the two MPK9 paralogs were differentiated in *H. annuus* and *C. morifolium*. MPK3 and MPK6 were considered to be closely related and functionally redundant in *A. thaliana*, and they were also well grouped. *H. annuus* had one more MPK6 than *A. thaliana*, indicating that MPK6 was duplicated after the speciation event between *A. thaliana* and *H. annuus*. In Group A, AtMPK11 and AtMPK13 lacked motif 7, which was found in the other members, possibly implying that these two members underwent changes in function relative to other members of Group A. MPK13 in Group B also appeared to show similar behavior to MPK6. MPK10 in Group A, MPK11/5/12 in Group B and MPK17 in Group D appeared only in *A. thaliana*, and the function of these genes has rarely been reported in plants, possibly suggesting that the gene was functionally redundant and was lost during evolutionary history.

To elucidate the phylogenetic relationship among the MKKs and infer the evolutionary history of the MKK family, we constructed a phylogenetic tree with high support rates using MKK members from *A. thaliana*, *H. annuus* and *C. morifolium*. The members divided well into four groups. There were only two paralogs of *C. morifolium* and *A. thaliana* in Group C, while *H. annuus* contained an additional gene, HanXRQChr12g0360031, that lacked motifs 5/8/3, suggesting that pseudogenization may have occurred. In addition, we found that there was a large-scale expansion of Group D in *A. thaliana*, while *C. morifolium* and *H. annuus* each retained one ortholog, and the motifs in *A. thaliana* were also differentiated, suggesting that they were more important for survival in *A. thaliana*.

### *CmMPK*s and *CmMKKs* are involved in plant growth and development

Many members of the MAPK cascade had been shown to participate in plant vegetative and reproductive growth (Xu & Zhang, 2015). In this study, we observed that the expression patterns of *CmMPK1*, *CmMPK3.1*, *CmMPK4.1*, *CmMPK4.2* and *CmMPK6* were similar in all organs of *C. morifolium* (Fig. 3A). With the maturation and senescence of leaves, the expression levels of those *CmMPKs* increased, while their expression in the root and stem remained at a relatively low level. These members were speculated to function in plant development. These CmMPKs interact with CmMKK2 and CmMKK4 in the yeast two-hybridization assay, and CmMPK3.1/6 interact with CmMKK9 (Fig. 6). Meanwhile, the expression patterns of *CmMKK9* and *CmMPK3.1/6* were similar (Fig. 3B). *CmMKK9* was hypothesized to regulate plant growth by the phosphorylation of CmMPK3.1, CmMPK4.1 and CmMPK6. However, the expression levels of CmMKK2 and CmMKK4 during development differed from those of CmMPK3.1, CmMPK4.1 and CmMPK6. The expression of CmMKK4 and CmMKK6 in various organs instead appeared constitutive (Fig. 3B). These proteins were speculated to provides important dose-effects in the MAPK cascade. Earlier studies had shown that MKK9 could activate MPK3/6 and that the activation of the CmMKK9-MPK3/6 cascade induced ethylene synthesis (Xu et al., 2008), which was consistent with the results of the yeast two-hybrid assay in this experiment. Another MAPK cascade of *A. thaliana,* composed of MKK4/5 and MPK3/6, was shown to promote local cell proliferation by promoting downstream RLKs to regulate inflorescence structures (Meng et al., 2012). Leaf senescence was delayed in plants with single mutations of *MKK9* or *MPK6*, and the overexpression of *MKK9* led to premature leaf senescence. This evidence demonstrated that the MKK9-MPK6 cascade was involved in the regulation of leaf senescence (Zhou et al., 2009). According to their expression patterns in *C. morifolium, CmMPK1* and these *CmMPKs* and *CmMKKs*, which had been previously shown to play a role in plant development, showed similar expression trends during plant development. CmMKK2 interacts with CmMPK3.1, CmMPK6 and CmMPK4.1 in the yeast two-hybrid system (Fig. 6) and had been shown to be upstream MKK of AtMPK4/6 in *A. thaliana* (Teige et al., 2004). This report was consistent with the results in this experiment, and CmMKK2 was also speculated to be involved in the regulation of plant development via the regulation of downstream MPKs.

### *CmMPKs* and *CmMKKs* were involved in response to temperature stresses

Most members of the MAPK cascade had been reported to show a response to temperature stress. The MAPK cascade pathway in *Brachypodium distachyon* was temperature sensitive: 90% of the MAPK cascade kinase genes were induced under cold stress, and 60% of the genes were induced by high temperature stress (Jiang et al., 2015). Most *Cucumis sativus CsMPK*s (except *CsMPK3*, *CsMPK7* and *CsMPK13*) were downregulated after cold treatment; however, most *CsMAPKs* were upregulated under heat shock stress except for *CsMPK3* and *CsMPK7* (Wang et al., 2015). The *A. thaliana* AtMEKK1-AtMAPKK2-AtMAPK4/AtMAPK6 pathway had been shown to play an important role in the defense against salt stress and cold stress (Teige et al., 2004). In contrast to *Cucumis sativus* (Wang et al., 2015), all *CmMPKs* except *CmMPK4.1* in *C. morifolium* were induced after cold treatment (Fig. 4A), but the expression level of *MPKs*, except *CmMPK4.1*, *CmMPK6* and *CmMPK13*, decreased or remained unchanged after heat shock treatment for 1 h (Fig. 4A), and it was hypothesized that the function of MPK differs among species. Experiments had shown that under the same stress conditions, some orthologs in *C. sativus*, *A. thaliana*, and *Oryza sativa* exhibit completely different expression patterns. For example, *AtMPK7* was significantly upregulated and *CsMPK7* was significantly downregulated under cold stress conditions. In addition, *OsMKK4* was upregulated under drought and cold stress conditions, while *CsMKK4* was downregulated under the same stress conditions (Wang et al., 2015). It was worth noting that the CmMKK9-CmMPK6 cascade had a consistent expression trend after temperature stress: *CmMKK9* (Fig. 4A) and *CmMPK6* (Fig. 4B) after cold and heat shock treatments, and we could surmise that this cascade may also be involved in the response to temperature stresses. It was noteworthy that only *CmMPK4.1* was increased in expression after heat-treatment for 1 h but not cold-induced, whereas its paralog, *CmMPK4.2*, was in the opposite. In *A. thaliana*, *AtMPK4* was reported to be a downstream component of H_2_S-associated cold stress, and both H_2_S and *MPK4* response to cold stress by regulate cold response genes and stomatal movement (Du et al., 2017). *CmMPK4.2* may play a similar role of *AtMPK4* in the cold stress. To date, there had been no report on the response of MPK4 to heat shock stress. We speculate that two CmMPK4 paralogs were functionally differentiated and participated in response to heat shock stress or cold stress in plants, respectively.

### *CmMPKs* and *CmMKKs* are involved in resistance to salt and osmotic stresses

Figure 3B shows that *CmMKK4* was maintained at a low and stable level in most tissues but specifically expressed in the root, and its expression was significantly increased after PEG and salt treatments (Fig. 5). Additionally, the overexpression of *ZmMKK4* in *A. thaliana* increased the tolerance to cold and salt stress relative to the control group by increasing the germination rate, lateral root number, plant viability, proline and soluble sugar content, chlorophyll and antioxidant enzyme activity (Kong et al., 2011). These facts suggest that *CmMKK4* is involved in the response to salt stress. Both *CmMPK13* and *CmMKK4* were induced by salt and osmotic stresses, and they were also shown to interact weakly in the yeast two-hybrid assay (Fig. 6). We could speculate that *CmMPK13* may be downstream of *CmMKK4* in the MAPK cascade and play a role in salt and osmotic stress. This MEKK1-MKK1/MKK2-MPK4 cascade had previously been shown to work together in experiments on the regulation of plant innate immunity (Gao et al., 2008; Kong et al., 2012) in addition to reactive oxygen signals and salicylic acid signals (Pitzschke et al., 2009). The expression patterns of *CmMPK4.2* and *CmMKK2* of this cascade in *C. morifolium* were similar (Fig. 5), and they were also shown to interact strongly in yeast (Fig. 6). The expression levels of *CmMPK4.2* and *CmMKK2* increased after salt stress and PEG treatment. Under salt stress or other abiotic stress, the dynamic balance of active oxygen is disturbed, and membrane lipid peroxidation or membrane lipid degreasing leads directly to increased active oxygen content in plants (Oukarroum et al., 2015). According to the above evidence, we surmise that the MKK2-MPK4 cascade is activated under salt stress in *C. morifolium* and regulates the reactive oxygen system to combat abiotic stress. The expression patterns of *CmMPK3.1* and *CmMPK3.2* were quite different from each other (Fig. 5). *CmMPK3.1* was not induced under abiotic stress treatment and exogenous hormone treatment, while the expression level of *CmMPK3.2* significantly increased after salt treatment. In addition, as shown in Fig. 3, *CmMPK3.1* expression in mature leaves and senescent leaves was significantly higher than in unexpanded leaves, while the expression of *CmMPK3.2* in every tissue remained at a very low level with no obvious features. Therefore, we surmised that the two *MPK3* paralogs in *C. morifolium* have functional differentiation: *CmMPK3.1* plays a role in plant development, while *CmMPK3.2* is involved in the salt stress response.

### *CmMPKs* and *CmMKK*s are involved in the resistance to exogenous phytohormone treatments

The relationship between MAPK cascades and ABA signaling had been previously reported (Danquah et al., 2014; De Zelicourt, Colcombet & Hirt, 2016). *MPK9* and *MPK12* had been reported to act as downstream genes of ROS to positively regulate guard cell ABA signaling (Jammes et al., 2009). The MAP3K17/18-MKK3-MPK1/2/7/14 cascade had been shown to be an intact ABA-activated MAPK cascade (Danquah et al., 2015). In this study, the *CmMPKs* and *CmMKKs* were quantitatively analyzed in GA- and ABA-treated *C. morifolium* (Fig. 5). The results revealed that the expression of *CmMPK1* did not change under exogenous GA or ABA treatment; the expression of *CmMKK3*, *CmMPK9.1* and *CmMPK9.2* increased slightly after exogenous ABA treatment; and the expression of *CmMPK18* increased most obviously after ABA treatment. MPK18 has been reported in relation to vascular-related functions in *A. thaliana* (Benhamman et al., 2017). Whether *MPK18* participates in the hormone response in *C. morifolium* needs further study.

### The interactions between CmMPKs and CmMKKs revealed by the yeast two-hybrid assay

In this study, the relationship between CmMPK and CmMKK in *C. morifolium* was examined by a yeast two-hybrid assay. CmMKK2 could interact strongly with CmMPK4/6 in the yeast two-hybrid system (Fig. 6), which was consistent with the composite cascade of AtMEKK1-AtMKK2-AtMPK4/AtMPK6 that had been demonstrated in *A. thaliana* (Teige et al., 2004). The yeast two-hybrid assay was also used in *A. thaliana* to gain insight into the potential relationships between all *A. thaliana* AtMPKs and their upstream activator AtMKKs (Lee et al., 2008): AtMKK2 interacts with AtMPK4, AtMPK6, AtMPK10, AtMPK11 and AtMPK13 in *A. thaliana*. In contrast to *A. thaliana*, CmMKK2 also interacts with CmMPK3 and interacts weakly with CmMPK1/13/18. AtMKK4 interacts strongly only with MPK3/6 in *A. thaliana* (Lee et al., 2008), whereas CmMKK4 exhibits a stronger yeast two-hybrid interaction than AtMKK4, and CmMKK4 not only strongly interacts with CmMPK3/6 but also shows a weak interaction with CmMPK1/4/13. In addition, AtMKK5, a paralogous MKK of AtMKK4, was the upstream gene of AtMPK3 and AtMPK6 in the MAPK cascade, and the AtMKK4/5-AtMPK3/6 cascade plays a key role in the response to many different stresses (Asai et al., 2002). Unlike AtMKK4, AtMKK5 interacts only with AtMPK6 in the yeast system (Lee et al., 2008). However, CmMKK5 not only interacts strongly with CmMPK6 but also interacts with CmMPK3 and interacts weak interaction with CmMPK1/4/13 (Fig. 6). Interestingly, this phenomenon was not limited to MKK5. AtMKK9 does not interact with AtMPK6 in yeast two-hybrid assays, but it can actively phosphorylate AtMPK6 and AtMPK20 in vitro (Lee et al., 2008), and CmMKK9 not only interacts strongly with CmMPK3/6 in yeast two-hybrid experiments but also interacts weakly with CmMPK1 (Fig. 6). This discrepancy may be due to the limitations of the yeast system.

In the yeast two-hybrid assay, the results of the interactions between MKKs and MPKs in *C. morifolium* were different from those in *A. thaliana*. First, we speculated that the functions of MPKs in *C. morifolium* and their orthologs in *A. thaliana* had been differentiated. Second, false-positive and false-negative interference occurred in the yeast two-hybrid assay. In the yeast two-hybrid assay, AtMKK4 interacts strongly only with AtMPK3/6, and AtMKK5 interacts only with AtMPK6 (Lee et al., 2008), but in the protein chip analysis, AtMKK4 was found to phosphorylate AtMPK3, AtMPK5, AtMPK6 and AtMPK10, while AtMKK5 seemed to be more promiscuous, phosphorylating AtMPK3, AtMPK4, At MPK5, AtMPK6, AtMPK8, AtMPK10 and AtMPK16 (Popescu et al., 2009). Thus, additional in vitro phosphorylation experiments on particular CmMKK and CmMPK interactions are needed.

## Conclusion

This study is the first transcriptome-wide analysis of the MPK and MKK family in *C. morifolium*. The transcription patterns of 11 *CmMPKs* and six *CmMKKs* were investigated in different organs of chrysanthemum, as well as in response to various phytohormones and abiotic stresses. In addition, the possibility that CmMKK4-CmMPK13 and CmMKK2-CmMPK4 may be involved in regulating salt resistance and the relationship between CmMKK9-CmMPK6 and temperature stress are first mentioned in this study. These findings lay the foundation for future research on the functions of *CmMPKs* and *CmMKKs* in plant stress response and growth, which will promote their application in chrysanthemum breeding.

## Supplemental Information

10.7717/peerj.5037/supp-1 Supplemental Information 1 Dataset 1. Sequences used in this manuscript.

10.7717/peerj.5037/supp-2 Supplemental Information 2 Table S1. Primers used in the manuscript.

10.7717/peerj.5037/supp-3 Supplemental Information 3 Table S2. Interactions between MKK and MPK revealed by yeast two-hybridization.

## Additional Information and Declarations

### Data Availability

The following information was supplied regarding data availability: The isolated 11 CmMPK (GenBank: MG334201–MG334212) and 6 CmMKK (GenBank: MG334196–MG334201) sequences are accessible via GenBank.
